# Towards an Improved LAI Collection Protocol via Simulated and Field-Based PAR Sensing

**DOI:** 10.3390/s16071092

**Published:** 2016-07-14

**Authors:** Wei Yao, David Kelbe, Martin van Leeuwen, Paul Romanczyk, Jan van Aardt

**Affiliations:** 1 Chester F. Carlson Center for Imaging Science, Rochester Institute of Technology, 54 Lomb Memorial Drive, Rochester, NY 14623, USA; wxy3806@rit.edu (W.Y.); dave.kelbe@gmail.com (D.K.); vanleeuwen.martin@gmail.com (M.v.L.); pavdpr@gmail.com (P.R.); 2 Department of Geography, University College London, Pearson Building, Gower Street, London WC1E 6BT, UK

**Keywords:** HyspIRI, AVIRIS, DIRSIG, leaf area index, photosynthetically active radiation

## Abstract

In support of NASA’s next-generation spectrometer—the Hyperspectral Infrared Imager (HyspIRI)—we are working towards assessing sub-pixel vegetation structure from imaging spectroscopy data. Of particular interest is Leaf Area Index (LAI), which is an informative, yet notoriously challenging parameter to efficiently measure in situ. While photosynthetically-active radiation (PAR) sensors have been validated for measuring crop LAI, there is limited literature on the efficacy of PAR-based LAI measurement in the forest environment. This study (i) validates PAR-based LAI measurement in forest environments, and (ii) proposes a suitable collection protocol, which balances efficiency with measurement variation, e.g., due to sun flecks and various-sized canopy gaps. A synthetic PAR sensor model was developed in the Digital Imaging and Remote Sensing Image Generation (DIRSIG) model and used to validate LAI measurement based on first-principles and explicitly-known leaf geometry. Simulated collection parameters were adjusted to empirically identify optimal collection protocols. These collection protocols were then validated in the field by correlating PAR-based LAI measurement to the normalized difference vegetation index (NDVI) extracted from the “classic” Airborne Visible Infrared Imaging Spectrometer (AVIRIS-C) data ($R^{2}$ was 0.61). The results indicate that our proposed collecting protocol is suitable for measuring the LAI of sparse forest (LAI < 3–5 ($m^{2}/m^{2}$)).

## 1. Introduction

The Hyperspectral Infrared Imager (HyspIRI) mission has been proposed by the Jet Propulsion Laboratory (JPL) in response to NASA’s Earth Science Decadal Survey [1]; this mission is geared to provide global imaging spectroscopy data to benefit research in domains ranging from coastal bathymetry to oceanography, wildfire science, volcanology, and terrestrial ecosystems. Although its 185 km swath width provides global coverage and 15-day revisit times, its large ground sample distance (GSD) introduces uncertainty in subpixel spectral variation. This is especially relevant in the open woodland forest environment, where trees are mixed with shrub, grass, bare soil, and rock. Our team participated in this mission by investigating the impact of sub-pixel structural variation on the assessment of vegetation structure via imaging spectroscopy data [2].

Vegetation structural parameters are related to the state and dynamics of the forest function, and therefore have important implications across domains. Of particular interest is Leaf Area Index (LAI), which is defined as the ratio of one-sided leaf area per unit ground area for flat broadleaf species [3]. An extended definition, i.e., the ratio of half of the total intercepting area per corresponding area on the ground, was proposed for all kinds of leaves, e.g., rolled leaves and needles of a coniferous tree [4,5]. LAI is a characteristic attribute in the description of the plant-atmosphere interface, and thus is a key input for models predicting variables such as ecosystem spatial distribution, health, photosynthesis, transpiration, and energy transfer [6,7,8]. However, accurate and precise measurement of LAI is notoriously challenging [9]. Destructive methods are time consuming and prohibitive in most practical settings [10]. Indirect field-based methods rely on measurements of canopy radiation transmission, yet their cost per area is high and uncertainty remains around an optimal sampling protocol [11]. Remote sensing techniques provide an alternative and much more cost effective approach, which is based on empirical relations between vegetation indices and LAI, yet its application still requires an effective sampling of in situ data [12,13]. The existing LAI retrieval techniques from passive remotely sensed images were reviewed and their capability was demonstrated when applied to different remote sensing devices by several recent studies [14,15]. These techniques could be applied to new imaging spectroscopy data, e.g., the proposed HyspIRI imaging spectroscopy space mission.

In typical research settings, sparse sets of field data, collected using terrestrial instruments, are used to calibrate and validate wall-to-wall Earth observation (EO) data acquired from airborne or orbital instruments. The majority of field-based methods for acquiring LAI utilize optical analysis of *gap fraction* and sometimes also the *gap size distribution* [9,11,16]. Gap fractions can be obtained from measurements of above- and below-canopy direct and diffuse radiation, which relaxes requirements on weather conditions. One such instrument of the latter type is the AccuPAR LP-80 Ceptometer (Decagon Devices, Inc., Pullman, WA, USA), used in this study, which calculates LAI from the ratio of above-canopy and below-canopy photosynthetically-active radiation (PAR) [17].

The AccuPAR LP-80 ceptometer has been validated for measuring the LAI of crops [18,19,20]; however, the sampling protocol and sensor deployment are crucial to obtaining an accurate LAI measurement [20,21]. Its capability for measuring forest LAI still needs to be fully explored [22,23], especially with regard to the marked differences in canopy size, spatial variability in canopy transmission and the required number of measurements or sampling density. Due to the variability among individual measurements, a suitable sampling protocol, which includes the sampling density and sensor deployment, is needed to obtain a reliable mean estimate within a forest plot. Thus, we identified a knowledge gap in terms of the appropriate field sampling protocols necessary to measure LAI of sparse woodland that is characterized by profound heterogeneity in leaf area density at the scale of a HyspIRI pixel. The sampling techniques for measuring other forest parameters, i.e., those related to even general forest inventory, have been fully discussed and could benefit from this study [24].

To summarize, the objectives of this research are to (i) validate the simulation of the specific AccuPAR LP-80 PAR sensor using Digital Imaging and Remote Sensing Image Generation (DIRSIG); (ii) determine the minimum collection parameters required to obtain a reliable mean estimate of LAI for a 80m×80m forest plot, as it relates to the context of HyspIRI-based assessment of LAI; and (iii) build a suitable regression model for estimating forest LAI from VIs derived from AVIRIS-C data. These outcomes will be used to address science questions related to the HyspIRI mission in follow-up studies, namely, the assessment of appropriate VIs to estimate sub-pixel vegetation structural parameters, e.g., LAI and canopy cover, from relatively coarse scale (30–60 m) HyspIRI data. Furthermore, the narrow-band VIs extracted from imaging spectroscopy data also will be compared to the broad-band VIs extracted from multispectral images to highlight the advantages of an imaging spectroscopy approach. All of these efforts are based on reliable in-field LAI measurements that now can be collected by adopting the optimal sampling protocol proposed by this paper.

## 2. Methods

**Figure 1:**
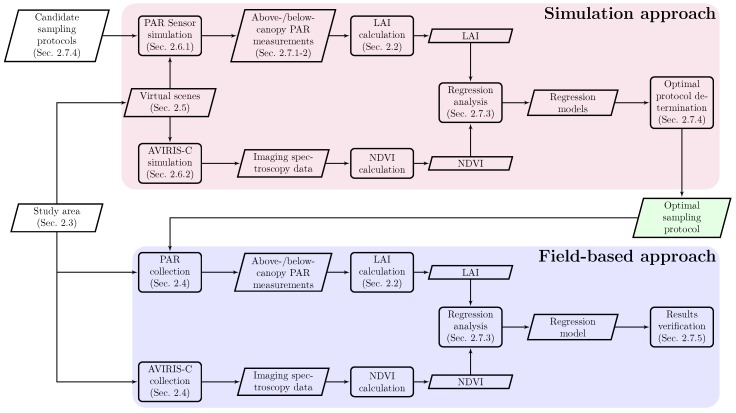
The study contains two parts: the simulation and field-based approach. Each step in the simulation environment is repeated in field-based approach to ensure that the simulations and models are correct.

A hybrid simulation/field-based approach was employed to negate time and monetary constraints associated with field deployments while providing absolute control over geometric and spectral reference data (Figure 1). The simulation approach (Figure 1, upper panel) utilized the DIRSIG model (Section 2.1). Two virtual DIRSIG scenes were developed (Section 2.5) for the study area (Section 2.3) using tree locations and diameters, extracted from field-measured diameter-at-breast-height (DBH) and stem maps for the center 20m×20m area (the National Ecological Observatory Network’s (NEON) plot) and tree locations from NEON’s airborne data for the larger plot. A virtual PAR sensor was then generated (Section 2.6.1) and validated for above- and below-canopy PAR measurement (Section 2.7.1 and Section 2.7.2). This was used to estimate LAI based on relevant theory (Section 2.2). Regression models were fit between estimated LAI from simulated PAR readings and simulated NDVI obtained from a synthetic AVIRIS-C sensor (Section 2.6.2) in order to validate the simulated PAR and LAI (Section 2.7.3) and determine the optimal PAR sampling protocol (Section 2.7.4).

These efforts were mirrored by a field-based approach (Figure 1, lower panel), which provided in situ reference data for the same study area (Section 2.3). The optimal PAR sampling protocol was used to inform field-based PAR measurement (Section 2.4), from which LAI was calculated and regressed against NDVI obtained from coincident AVIRIS-C spectroscopy data (Section 2.4). This provided additional verification of the proposed approach (Section 2.7.5).

### 2.1. DIRSIG Background

DIRSIG is a physics-based, first-principles radiometric modeling environment for the creation of synthetic remote sensing imagery that is radiometrically, geometrically, and temporally accurate [25]. The model is designed to generate passive broad-band, multispectral, imaging spectroscopy, low-light, polarized, active laser radar, and synthetic aperture radar datasets [26,27,28] through the integration of a suite of first-principles-based radiation propagation modules.

### 2.2. A Description of the Relevant LAI Theory

PAR is defined as the radiance in the wavelength range 400–700 nm, i.e., the visible light, which is absorbed by leaves during the photosynthesis process. Therefore, the amount of absorbed PAR could be used to estimate the LAI, as these two quantities are directly related. Monsi and Saeki [29,30] proposed an equation that is similar to Beer’s Law for constructing the connection between the ratio of the below-canopy irradiance, $E_{c}$, and the incident irradiance, $E_{0}$, and leaf area index, LAI. Norman and Jarvis [31] proposed a complete radiation penetration model because Monsi and Saeki’s equation is not accurate and suitable for all kinds of canopies. This approach was deemed not suitable for computation, due to the complexity. A simplified version was presented by Norman and Campbell [32]:

(1) $$\frac{E_{c}}{E_{0}}=\exp\left[\frac{A\cdot (1-0.47\cdot f_{b})\cdot LAI}{\left(1-\frac{1}{2\cdot K}\right)\cdot f_{b}-1}\right]$$

where $f_{b}$ is the fraction of direct beam to total incident PAR, and *A* is a constant equal to $0.283+0.785a-0.159a^{2}$, where *a* is the leaf absorptivity in the PAR band. Campbell proposed a form of K for all kinds of leaf angle distribution [33]:

(2) $$K=\frac{\sqrt{\chi^{2}+\tan^{2}\theta }}{1.47+0.450\chi +0.1223\chi^{2}-0.0130\chi^{3}+0.000509\chi^{4}}$$

where *χ* is the leaf angle distribution parameter. It is defined as the ratio of the horizontal semi-axis length to the vertical semi-axis length of the spheroid, described by the leaf angle distribution of a canopy. The value of *χ* is in the range of [0.1, 10]. The LAI can be calculated by solving Equation (1) when we have above-canopy PAR measurement, $E_{0}$, and below-canopy PAR measurement, $E_{c}$.

### 2.3. Study Areas

**Figure 2:**
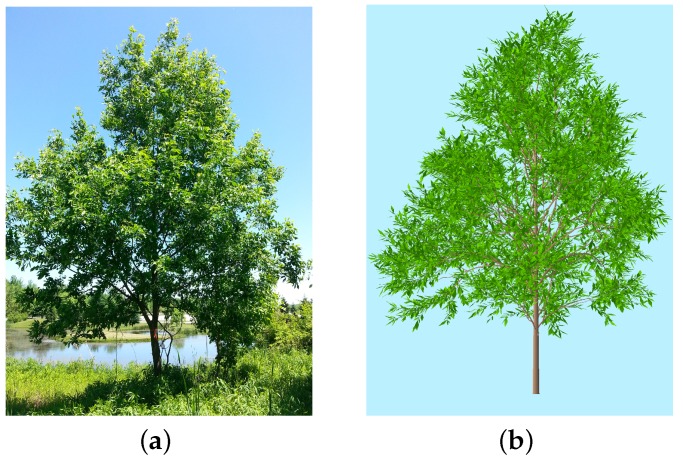
(**a**) an ash tree located within the Rochester Institute of Technology (RIT) campus and (**b**) its 3D model.

Two study areas were selected. The first area is a small grass field with an ash tree on the campus of Rochester Institute of Technology (RIT) (Figure 2a, $43^{\circ }05^{\prime }16.97^{\prime \prime }$ N, $77^{\circ }40^{\prime }49.04^{\prime \prime }$ W). Table 1 gives the structural parameters of the ash tree. This area was used to investigate the leaf area of a single tree.

**Figure 3:**
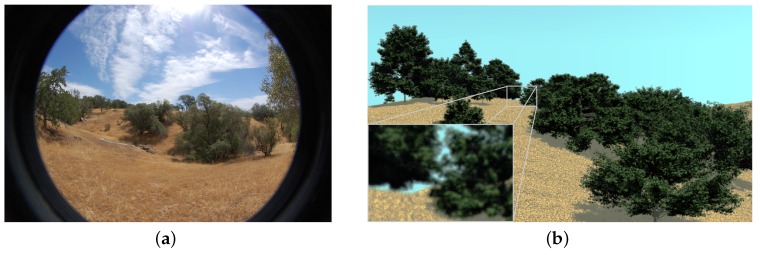
(**a**) plot #116 in the National Ecological Observatory Network’s (NEON) D17 Domain and (**b**) the virtual scene.

The second area is NEON’s Domain D17 (Pacific Southwest) located in San Joaquin Experimental Range (SJER), California, USA. The San Joaquin Experimental Range is an oak savanna site (Figure 3a, $37^{\circ }06^{\prime }43.77^{\prime \prime }$ N, $119^{\circ }44^{\prime }11.85^{\prime \prime }$ W). The dominating species are blue oak (*Quercus douglasii*), interior live oak (*Quercus wislizeni*), and grey pine (*Pinus sabiniana*) [34]. The NEON Airborne Observation Platform (AOP) team selected 20 observation plots in SJER. They measured the vegetation structural data in a 20m×20m area. Our field team enlarged three plots (#116, #36, and #824) into an 80m×80m area, which is slightly larger than the spatial resolution of HyspIRI (60 m). Multiple types of measurement were collected in those plots (see Section 2.4 for details).

### 2.4. Field Inventory and Airborne Imagery Data

The field data were obtained to support the construction of virtual scenes and the validation of simulation results as follows: During the summer 2013 collection, terrestrial laser scanner (TLS) data and the spectra of leaf, bark, and grass were collected in the study area to support virtual scene construction. Reference PAR measurements were collected using AccuPAR LP-80 instruments at plots #116, #36, and #824 in October 2014 according to the optimal collection protocol proposed in this paper for validation. The PAR data of the ash tree were collected using AccuPAR LP-80 instruments on RIT’s campus on 31 May 2014. The imaging spectroscopy data were collected by AVIRIS-C on 12 June 2013 and 6 October 2014 at approximately 15 m spatial resolution (dependent on topography). An Optech Gemini small-footprint waveform-recording LiDAR (Vaughan, Ontario, Canada) was operated onboard NEON’s aircraft to collect airborne LiDAR data on 13 June 2013 [35].

### 2.5. Virtual Scene Development

Two virtual scenes were constructed for this study. The 3D tree models were created in OnyxTREE [36] (Version 7.0, Onyx Computing, Inc., Cambridge, MA, USA) and matched the height, crown size, and leaf and bark optical properties of the field-measured trees. It should be noted that the number of branches and leaves of the models might be different from that of the actual trees. However, the virtual scene provides for full knowledge of vegetation-structural attributes, on a per-tree basis, for which AccuPAR readings can be derived and analyzed.

The first scene was based on the ash tree on the campus of RIT (Figure 2b). Both above-canopy and below-canopy PAR of the single crown were simulated. The second scene was based on plot #116 within the SJER (Figure 3b). There are 36 trees with crown diameters >2 m in the 80m×80m area. Twenty-six tree models (some models have multiple instances) were created by OnyxTREE and placed on the virtual terrain according to their real positions extracted from airborne LiDAR data. Table 2 gives a list of the geometrical size and positions of tree models in the plot #116 scene. The optical properties of tree bark, tree leaves, and ground used in the virtual scene were collected by our field team and NEON AOP team [35]. We used this scene to (i) investigate the LAI of a forest environment and (ii) determine the proper number of below-canopy PAR readings required to properly represent within-pixel variation, which is expressed at the pixel-level, i.e., at the 30–60 m spatial scale when collecting field data.

### 2.6. DIRSIG Simulation Design

#### 2.6.1. Development of a Simulated PAR Sensor

DIRSIG is typically used to simulate remote sensing devices such as a multispectral imagers, imaging spectrometers, or LiDAR instruments. A PAR sensor was simulated by DIRSIG for the first time in this study. The irradiance, *E*, from the hemispherical sky was simulated to reproduce the method of Norman and Campbell [32] in DIRSIG. The incident radiance, *L*, to the detectors was returned by DIRSIG. The relationship between radiance and irradiance is defined as:

(3) $$L=\frac{\;dE}{\;d\Omega \cos\theta }\;$$

where dE is the irradiance from the small solid angle, dΩ, which is mapped to the detector cell, and *θ* is the angle between irradiance and the normal of the detector surface. We rearranged Equation (3) and applied integration to both sides to obtain the irradiance from the whole hemispherical sky:

(4) ∫dE=∫LdΩcosθ⟹E=∫LcosθdΩ

Mathematical integration is achieved by summation in the discrete simulation:

(5) $$E≃\sum_{i}L_{i}\;\cos\theta_{i}\;\Omega_{i}$$

**Figure 4:**
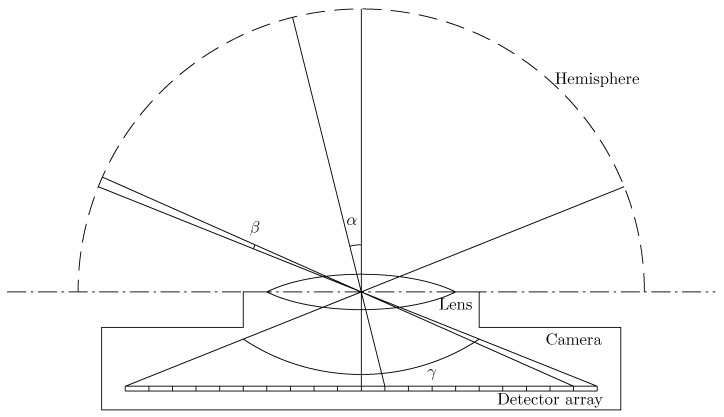
The angles mapped to the detector cells are not constant.

DIRSIG usually provides a 1D or 2D grid detector array and returns the radiance, $L_{i}$, captured by each detector cell, since this is the generic geometry of an imaging spectrometer. This type of detector array is not appropriate for this study for two reasons: (i) the angle of each detector cell is not a constant. As Figure 4 shows, the center detector cell maps to a larger angle than the cell on the periphery (α>β); and (ii) the normal 2D grid detector array cannot map the whole hemisphere because the angle *γ* cannot be $180^{\circ }$.

**Figure 5:**
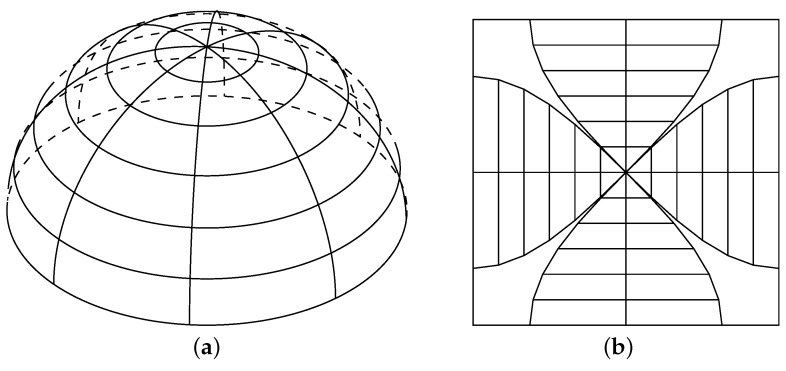
Flatten the hemisphere (**a**) into a plane (**b**).

DIRSIG provides another method, called “data-driven detector”, which allows users to define an arbitrary detector array other than the detector array on a grid. A user can set the pointing direction of a cell and the angular instantaneous field-of-view (IFOV). The hemisphere then is divided into tiny sections, with the same area corresponding to a detector cell. Each cell is mapped to the same solid angle. In this way, the hemisphere is flattened into a plane (Figure 5).

**Figure 6:**
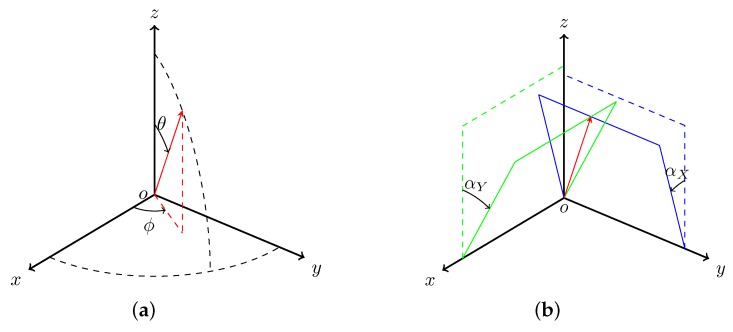
Convert (**a**) *θ* and *ϕ* in the spherical coordinate system to (**b**) $\alpha_{X}$ and $\alpha_{Y}$ in the *X*/*Y* angle system.

The spherical coordinate system is employed when the hemisphere has been flattened (Figure 6a). However, DIRSIG uses the *X*/*Y* angle relative to the optical axis (Figure 6b) to describe the pointing direction, since this *X*/*Y* angle-based coordinate system facilitates the definition of the pointing angle of a 2D grid detector array. The converting functions are given as:

(6) $$\begin{array}{c} \alpha_{X}=\tan^{-1}\left[\cos\left[\phi \right]\tan\left[\theta \right]\right], \end{array}$$

(7) $$\begin{array}{c} \alpha_{Y}=\tan^{-1}\left[\sin\left[\phi \right]\tan\left[\theta \right]\right]. \end{array}$$

Since the solid angles mapped to every detector cell are equal, and their integral is 2π, we get

(8) $$\Omega_{1}=\Omega_{2}=\cdots =\Omega_{N}=\frac{2\pi }{N}$$

where *N* is the number of the detector cells. Equation (5) can be simplified by substituting $\Omega_{i}$ with Equation (8):

(9) $$E≃2\pi \;\frac{\sum_{i=1}^{N}L_{i}\cos\theta_{i}}{N}$$

The size of the detector array is the next parameter that needs to be determined. As the Sun is the dominant source of downwelling radiance, the IFOV should be less than or equal to the size of the Sun to ensure that the Sun will be fully captured. The equatorial diameter of the Sun is $1.392\times 10^{6}$ km and the mean distance from the Earth is $1.496\times 10^{8}$ km [37]. When being observed from the Earth, the average size of the sun disk in radians is:

(10) $$\frac{1.392\times 10^{6}}{1.496\times 10^{8}}\approx 9.3\times 10^{-3}\left(\text{rad}\right)$$

Therefore, if a resolution of 350×350 pixel is selected, the IFOV is:

(11) $$\frac{\pi }{350}\approx 9.0\times 10^{-3}\left(\text{rad}\right)$$

**Figure 7:**
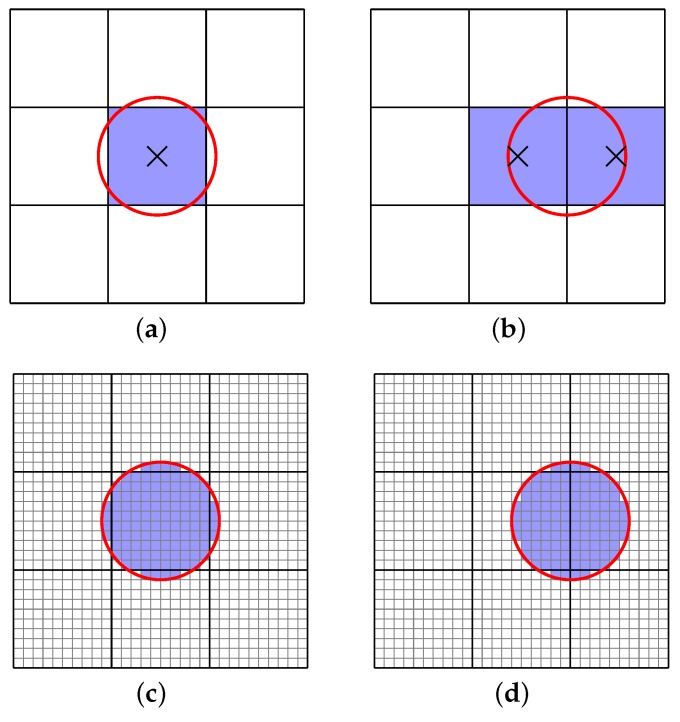
Capturing the sun disk with different spatial resolutions. (**a**) the Sun is mapped to one pixel; (**b**) the Sun is mapped to two pixels; (**c**) the same Sun position as in the above figure on a higher resolution detector array; and (**d**) the same Sun position as in the above figure on a higher resolution detector array.

However, this is only the minimal requirement. The Sun might be mapped to one or two pixel(s) in this resolution (Figure 7a,b). The solution to this problem is to decrease the IFOV (Figure 7c,d). If the resolution of the whole detector array is increased, more computing resources and longer simulation times will be required. However, areas of the sky outside the sun disk do not require such a high spatial resolution simulation. Therefore, a second virtual detector array is introduced, which points toward the Sun [38] and collects the radiance from the Sun and a small surrounding area in a small IFOV ($4.5\times 10^{-4}$ (rad)).

#### 2.6.2. Simulation of AVIRIS-C Sensor

DIRSIG has been developed and validated [26,27] as a remote sensing device simulator; therefore, it proved to be straightforward to simulate the AVIRIS-C sensor within DIRSIG. We configured DIRSIG to mimic the AVIRIS-C sensor parameters as closely as possible, with Table 3 providing the key configurations. The prevailing atmosphere was simulated via MODTRAN4 (Version 3 Revision 1, Spectral Sciences, Inc., Burlington, MA, USA and Air Force Research Laboratory, Hanscom AFB, MA, USA) [39].

### 2.7. Experiment Design

#### 2.7.1. Experiment 1a: Validating Simulated Above-Canopy PAR

We simulated the collection of above-canopy PAR with DIRSIG as the first step. When simulating the above-canopy PAR collection, we only need to consider the direct radiance from the Sun and the radiance scattered by the atmosphere. Above-canopy PAR data were collected in SJER on 13 June 2013 from 7h00 to 17h00 (8h00–18h00 in daylight saving time) at one-minute intervals. A series of simulations were performed to reproduce the collection parameters and generate corresponding synthetic PAR data.

#### 2.7.2. Experiment 1b: Validating Simulated PAR and LAI for a Single Crown

**Figure 8:**
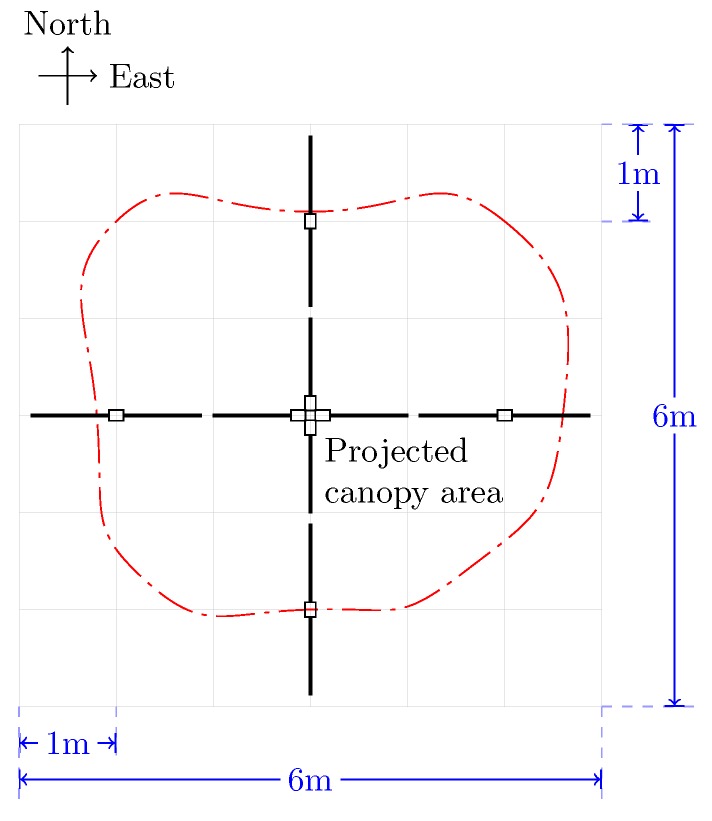
Using AccuPAR LP-80 to collect the below-canopy photosynthetically-active radiation (PAR) reading along quadrantal directions.

We then simulated the collection of below-canopy PAR of the ash tree on RIT’s campus. The virtual PAR sensor was placed on a 0.1 m interval grid around the tree model to collect the below-canopy PAR readings. The sampling interval of 0.1 m was selected in order to reproduce the simulation results as closely as possible to those collected using the LP-80 ceptometer (Decagon Devices, Inc., Pullman, WA, USA). The LP-80 ceptometer returns eight PAR measurements, corresponding to eight segments from a probe of 0.8 m in length, i.e., the length of each segment is 0.1 m. The spatially-explicit LAI for this single crown was calculated as individual point-based samples, i.e., the LAI for every 0.1×0.1m square inside the projected crown area. In order to validate the simulated LAI measurements, actual field measurements were taken both below the tree canopy and outside the extent of the ash tree, i.e., open-sky, using LP-80 instruments. Such a single-tree approach is atypical of how LAI is assessed from a traditional perspective, namely within forests. However, this best-case scenario enabled us to examine our simulation model in a simpler environment and limit the complexity before extending the simulation to our more complex forest scene. Figure 8 gives the collecting scheme. There were three AccuPAR measurements along each direction. These measurements then were compared to the simulated data obtained from the corresponding virtual tree (Figure 2b).

#### 2.7.3. Experiment 1c: Validating Estimated LAI from Simulated PAR for the Forest Site Using Regression to Model NDVI

Following the previous step, we simulated the PAR sensor in the forest scene. We were not able to validate simulated PAR due to expected discrepancies between the virtual scenes and their real counterparts, e.g., the modeled trees were not identical, but similar to the real ones, and small trees (crown diameter < 2 m) were ignored when the virtual scene was constructed (see Section 2.5 for details). As an alternative, we investigated the relationship between LAI, calculated from PAR, and the normalized difference vegetation index (NDVI), extracted from (hyperspectral) imagery. NDVI is widely used to assess LAI. Previous studies showed that the NDVI–LAI model had a strong linear relationship at low LAI values (LAI < 3–5 or NDVI < 0.8) [12,13,40,41]. Therefore, we extracted the NDVI from synthetic imaging data, obtained from an AVIRIS-like virtual sensor, and compared it to LAI obtained from the simulated PAR measurements to generate a linear model, since our study area had a relatively low LAI. The spatial resolution of the simulated AVIRIS-C data is 15 m, which is consistent with the real AVIRIS-C data collected in our study area in 2013 and 2014. The NEON plot #116 was divided into 25 squares of 15m×15m each. The total effective area is 75m×75m, which is slightly less than the plot area (80×80 m). All of the LAI measurements within a 15m×15m square were averaged as the LAI of a pixel. Then, the regression model of NDVI and LAI was created at the pixel level. Although NDVI is known to have limitations, such as saturation at high LAI levels [42,43], it provides an opportunity to assess correlations between an established narrow-band index and our virtual LAI.

#### 2.7.4. Experiment 2a: Determining Optimal Spacing by Comparing PAR to NDVI for Forest Sites at Various Intervals

It is common practice to obtain LAI measurement along several parallel transects for forest environments [24]. We proposed three sampling protocols with gradually varied intervals between parallel transects. The PAR measurements were collected at each meter along a transect because the length of the AccuPAR LP-80’s wand is around 1 m. Table 4 gives the intervals and required number of measurements.

The LAI–NDVI regression models were created from simulation results for each protocol. The models were compared and provided an opportunity to assess the optimal sample spacing for field data collection, in order to achieve adequate mean estimates, given the variability within an 80m×80m forest plot. The previous study showed that a linear relationship between LAI and NDVI exists for low canopy cover (LAI) forests. Therefore, we considered the LAI estimates as being accurate if a model was similar or close to the theoretical model. A Student’s *t*-test was used in the study to determine if two linear models are similar [44]. The test statistic for the slopes is:

(12) $$T_{b}=\frac{b_{1}-b_{2}}{S_{b_{1}-b_{2}}}$$

where $b_{1}$ and $b_{2}$ are the slopes of two models and $S_{b_{1}-b_{2}}$ is the standard deviation of the estimated difference between slopes

(13) $$S_{b_{1}-b_{2}}=S_{P,Y|X}\sqrt{\frac{1}{\left(N_{1}-1\right)S_{X_{1}}^{2}}+\frac{1}{\left(N_{2}-1\right)S_{X_{2}}^{2}}}$$

The test statistic for the intercept is:

(14) $$T_{a}=\frac{a_{1}-a_{2}}{S_{a_{1}-a_{2}}}$$

where $a_{1}$ and $a_{2}$ are the intercepts of two models, and $S_{a_{1}-a_{2}}$ is the standard deviation of the estimated difference between intercepts:

(15) $$S_{a_{1}-a_{2}}=S_{P,Y|X}\sqrt{\frac{1}{N_{1}}+\frac{1}{N_{2}}+\frac{{\bar{X}}_{1}^{2}}{\left(N_{1}-1\right)S_{X_{1}}^{2}}+\frac{{\bar{X}}_{2}^{2}}{\left(N_{2}-1\right)S_{X_{2}}^{2}}}$$

Whether or not the decisions of two models are considered similar is dependent on the significance level, *α*, which is usually set to be 0.01, 0.05 or 0.10 for a two-tailed test [45]. A significance level of 0.05 is the most common value in most statistical textbooks, e.g., [44,45]. However, the test results based on the significance level do not tell us that the models are absolutely different from each other, but rather indicate how confidently we can state that they are not the same. Therefore, there is no definitive *α* value. We opted for a less conservative *α* value of 0.1, implying that we can be incorrect 10% of the time.

#### 2.7.5. Experiment 2b: Comparing in Situ LAI Estimates to NDVI to Verify Simulated Results

Finally, our field team revisited plot #116 and two additional plots (#36 and #824) in the SJER site to collect in situ LAI measurements with the identified optimal sampling protocol, after which a regression model with the NDVI extracted from actual AVIRIS-C data was constructed. The regression model for real data was compared to the model of simulated data to validate our simulation results.

## 3. Results and Discussion

### 3.1. Experiment 1a: Simulated Above-Canopy PAR

**Figure 9:**
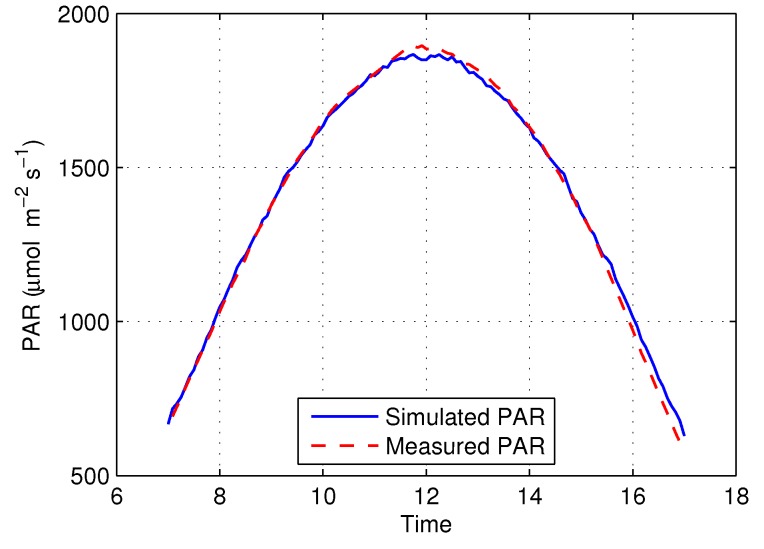
The simulated PAR and measured above-canopy PAR on San Joaquin Experimental Range (SJER).

Figure 9 shows the simulated and actual above-canopy PAR measurements obtained from NEON plot #116, with a correlation coefficient of $R^{2}=0.998$ and root-mean-square error of RMSE = 23.94 (μmol·m${}^{-2}$·s${}^{-1}$).

**Figure 10:**
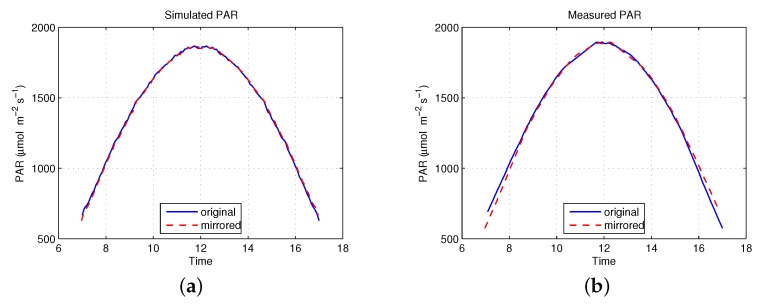
An example of the original and horizontally mirrored PAR: (**a**) simulated PAR and (**b**) measured PAR.

It is evident from Figure 9 that PAR peaks at 12h00 (noon) due to the angular projection effects of the Sun’s flux on the horizontal detector element. However, the simulated PAR is slightly larger than the measured PAR during the late afternoon. The PAR curve should be symmetric in theory. Therefore, if the curve is replicated horizontally, it should overlap with the original, Sun-derived curve. Both the simulated and measured PAR were mirrored and compared to their original curves in order to determine which curve might be problematic. Figure 10 shows that the simulated PAR perfectly overlaps with the non-mirrored curve, but the measured PAR does not. There are two likely reasons: (i) the atmospheric conditions changed during the day, for example, vapor concentration is higher in the morning than in the afternoon; and (ii) the error might occur because the AccuPAR LP-80 Ceptometer was not perfectly leveled during collection. These are typical examples of two types of errors in this study: One type of error relates to modeling errors, which were introduced by the assumptions to reduce the complexity of the model and discretization for the mathematical model that makes computational analysis possible [46]. The other type is an example of an observational error, i.e., the difference between a measured value and its true value. The RMSE of above-canopy simulation results was 23.94 (μmol·m${}^{-2}$·s${}^{-1}$), which is quite small; hence, we concluded that we could safely omit the effects of the errors.

### 3.2. Experiment 1b: Simulated PAR and LAI for a Single Crown

**Figure 11:**
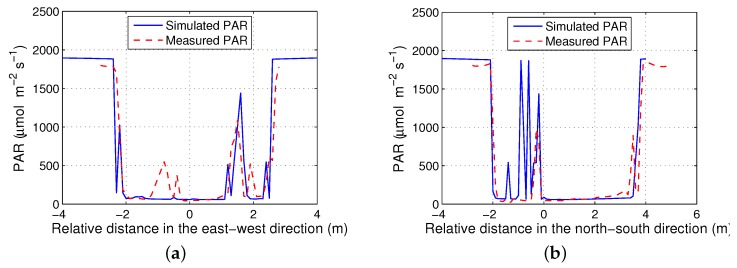
The simulated and measured below-canopy PAR for the RIT Ash Tree: (**a**) along the east–west direction; (**b**) along the north–south direction.

**Figure 12:**
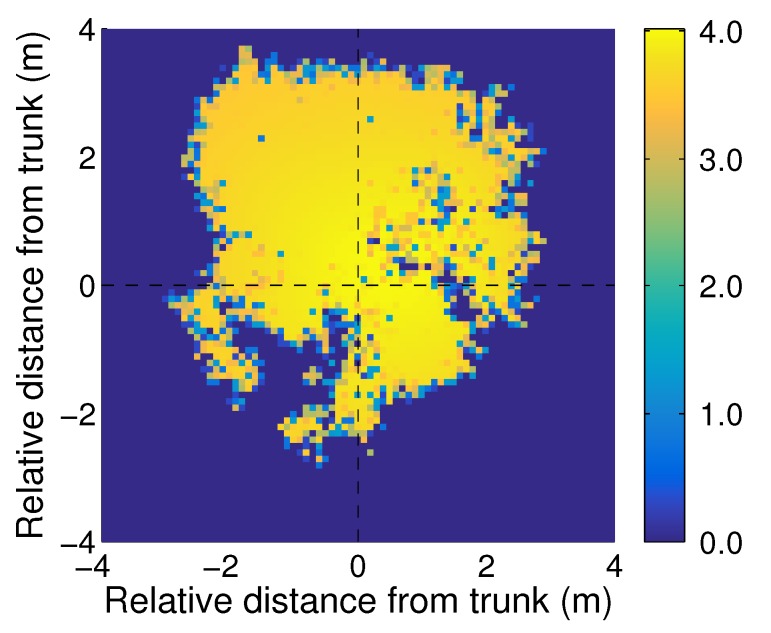
The leaf area calculated by simulation results of the model tree.

Figure 11 shows the comparison of simulated and measured below-canopy PAR for the ash tree on RIT’s campus. The position of the ash tree was set the same as the original, and the PAR was captured along quadrantal directions (the dashed lines in Figure 12). Figure 11 shows the measured and simulated below-canopy PAR, with correlation coefficients of $R^{2}=0.706$ (along the east–west direction, Figure 11a) and $R^{2}=0.786$ (along north–south direction, Figure 11b). Spikes in the plot are due to sun flecks, i.e., where sunlight reaches the detector directly through gaps in the canopy. Note that these PAR features did not directly overlap between simulated and actual data, due to differences in tree structure between the modeled (virtual) tree and the real tree.

Below-canopy PAR measurements subsequently were normalized by the above-canopy PAR, collected at the same time, to produce a high-resolution spatial map of LAI distribution (Figure 12). As would be expected, we found that the LAI readings were more uniform in the center of the shadow and that there were some locations with very low LAI, due to the gaps in the canopy. The size of the largest gap was about 1 m, which was larger than the length of the AccuPAR probe (≈0.8 m). It thus was concluded that, for a single tree of the size, shape, and species type used in this study, multiple field-based PAR measurements along a transect within the shadow of such a tree will be required. However, future efforts will include a more diverse set of trees in terms of spectral and structural characteristics.

### 3.3. Experiment 1c: Estimated LAI from Simulated PAR for the Forest Site Using Regression to Model NDVI

**Figure 13:**
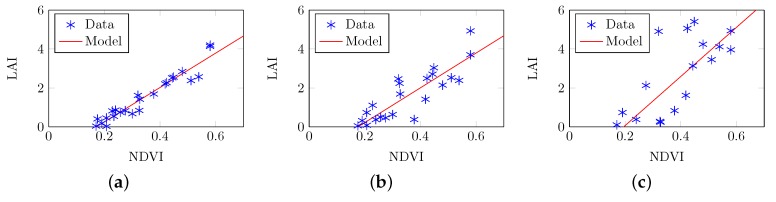
Leaf area index (LAI) estimates derived from simulated AccuPAR readings against the normalized difference vegetation index (NDVI) extracted from simulated Airborne Visible/Infrared Imaging Spectrometer (AVIRIS) images. (**a**) 45 PAR readings were simulated along three transects in each 15m×15m square ($R^{2}=0.92$, RMSE=0.33); (**b**) 30 PAR readings were simulated along two transects in each 15m×15m square ($R^{2}=0.77$, RMSE=0.66); and (**c**) 15 PAR readings were simulated along one transect in each 15m×15m square ($R^{2}=0.66$, RMSE=1.24).

The comparison of simulated, forest-level LAI and NDVI, as a representative narrow-band index, was achieved via three setups with increasing spatial frequency, as discussed in Section 2.7.4. Figure 13 shows the simulated forest LAI vs. NDVI at 5 m transect spacing ($R^{2}=0.92$, RMSE=0.33), at 10 m transect spacing ($R^{2}=0.77$, RMSE=0.66), and at 15 m transect spacing ($R^{2}=0.66$, RMSE=1.24). The linear models of LAI vs. NDVI are listed below:

(16) $$\begin{array}{cc} \mathrm{LAI} & =8.826\times \mathrm{NDVI}-1.506\;\;(\text{for}\;\text{spacing}\;=\;5\;m) \end{array}$$

(17) $$\begin{array}{cc} \mathrm{LAI} & =8.928\times \mathrm{NDVI}-1.566\;\;(\text{for}\;\text{spacing}\;=\;10\;m) \end{array}$$

(18) $$\begin{array}{cc} \mathrm{LAI} & =12.61\times \mathrm{NDVI}-2.457\;\;(\text{for}\;\text{spacing}\;=\;15\;m) \end{array}$$

### 3.4. Experiment 2a: Optimal Spacing by Comparing PAR to NDVI for the Forest Site at Various Intervals

The 5 m transect provided solid results but came at the cost of being time-consuming for practical collection in the field. Table 5 gives the *t*-test results for linear model comparison. The results showed that the models were similar at an *α* value of 0.1 for the 10 m- and 5 m-interval, while they were different for the 15 m- vs. 5 m-interval. If we compare the *p*-values of the two tests, we find that 0.059 is distinctly smaller than the *p*-value of 0.928, which also indicates that the 10 m- and 5 m-interval models have similar slopes, while the 15 m- and 5 m-interval models do not. The linear model for the best LAI estimates (5 m-interval) could be considered as the true model ($R^{2}=0.92$); however, the model for the 10 m-interval is still acceptable because it is not statistically different from the 5 m-interval model. The linear model for the 15 m-interval, on the other hand, is not acceptable because of its statistical difference from the 5 m-interval model. The $R^{2}$ fell by approximately 15% for the 10 m-interval and the 5 m-interval scenarios; however, only half the number of measurements were required in the case of the 10 m spacing scenario (Table 4). The 10 m interval, therefore, was deemed as being an appropriate sampling protocol, since that spacing balances efficiency with accuracy and precision. The results will contribute to our own and other studies’ efforts to better evaluate the effects of within-pixel structural variability on coarse spatial resolution imaging spectroscopy spectra.

### 3.5. Experiment 2b: Comparing in Situ LAI Estimates to NDVI to Verify Simulated Results

**Figure 14:**
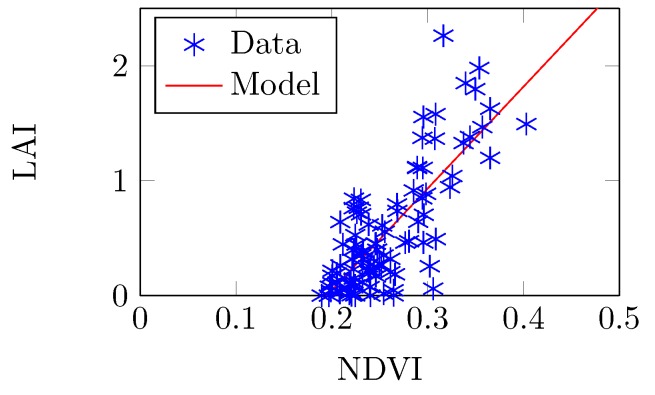
NDVI extracted from a real AVIRIS-C image was used to verify the in situ forest LAI, where $R^{2}=0.61$, RMSE=0.34.

The LAI–NDVI model of optimal sampling protocol (Section 2.7.4) was confirmed via a field effort where we collected LAI (AccuPAR LP-80) based on 10 m-interval transects in three 80m×80m plots of SJER on 5–7 October 2014 and verified results with NDVI extracted from AVIRIS-C data (Figure 14). The obtained linear model was

(19) LAI=8.858×NDVI-1.725

which is consistent with the simulation model (Equation (17)) because the *t*-test results of slope comparison are T=0.0623,p=0.950, and the *t*-test results of intercept comparison are T=0.471,p=0.538. Both *p*-values were larger than α=0.10. When we estimated LAI from NDVI by using the two models (Equations (17) and (19)), the RMSE between the two groups of LAI was 0.177, which we considered a negligible difference. The obtained coefficient of determination ($R^{2}=0.61$) was lower than the simulation result, but was still slightly higher than regression models reported in other papers and previous efforts, e.g., $R^{2}=0.53$ [13] and $R^{2}=0.55$ [41]. The discrepancy in $R^{2}$ values between the simulation and field-based, actual data results was attributed to our inability to accurately simulate the true structural variability in a natural forest, although the LAI vs. NDVI trend in both the simulation and real scenarios matched very well. As such, it was concluded that the simulation approach enabled us to: (i) accurately model LAI collection behavior when using a PAR sensor, such as the AccuPAR LP-80; (ii) that the identified LAI collection protocol resulted in the best trade-off between accuracy (and precision) and resources; and (iii) enabled us to advance the scientific inquiry into sub-pixel structural impacts on imaging spectrometer data by clearly establishing rules for field collection protocols.

## 4. Conclusions

In an effort to understand the impact of sub-pixel structural variation on large-footprint imaging spectroscopy, e.g., as obtained from the envisioned HyspIRI mission, a simulation approach was used to provide absolute knowledge of field-level target geometry and associated platform-based radiometry. We presented a simulation approach for measuring one structural metric of interest, LAI, using the fractional PAR ratio approach [17,31]. This approach was validated with field data obtained from AccuPAR LP-80 measurements ($R^{2}=0.706\;\text{and}\;0.786$) and subsequently compared to LAI vs. NDVI modeling using data obtained from simulated AVIRIS imagery. An appropriate sampling protocol for LAI data collection was proposed at 10 m transect spacing, which ensured efficient data collection. We recognize that a higher density model, such as the 5 m spacing, would result in more accurate and precise LAI estimates. However, given the fact that the 5 m model and 10 m model were similar in slope and intercept, i.e., the model form is similar, we recommend the 10 m field sampling approach. This is due to its reduction in resource requirements (time and money) at a limited loss in model performance. Finally, these simulation results were validated using real in-field LAI measurements at the defined transect spacing, along with AVIRIS-C airborne imaging spectroscopy data ($R^{2}=0.61$). The slight discrepancy between simulation and real results was attributed to our inherent inability to truly mimic the structural variability present in nature, although we did conclude that trends between the two approaches were similar as far as regression results were concerned.

We further concluded that (i) an in-field PAR sensor can be simulated in the DIRSIG model and (ii) a Ceptometer, like the AccuPAR LP-80, is suitable to collect the LAI of an open forest (LAI < 3–5), when an appropriate sampling protocol is selected. Recommendations for future efforts include (i) simulating the PAR sensor in other virtual scenes, such as a dense forest scene and (ii) determining the capability of the Ceptometer and a befitting protocol for a dense forest scene (LAI > 3–5).

## Figures and Tables

**Table 1:** The structural parameters of the ash tree.

Parameter	Value (Unit)
Height	7.4 (m)
Crown width (in West-East direction)	4.8 (m)
Crown width (in South-North direction)	4.1 (m)
DBH (at the first branch, 1.2 m from ground)	18 (cm)
LAI	3.5

DBH: diameter at breast height; LAI: leaf area index.

**Table 2:** List of tree models in the plot #116 scene (unit: meter). The positions are in scene East North Up (ENU) coordinates from the center of the plot ($37^{\circ }06^{\prime }43.77^{\prime \prime }$ N, $119^{\circ }44^{\prime }11.85^{\prime \prime }$ W).

ID	Type	Height	Crown Dia.	Instance 1		Instance 2		Instance 3	
				*x*	*y*	*x*	*y*	*x*	*y*
1	Broadleaf	9.78	17.34	2.96	−15.16	–	–	–	–
2	Broadleaf	10.87	11.82	5.76	−2.86	–	–	–	–
3-1	Conifer	13.16	12.66	−7.67	−9.15	–	–	–	–
3-2	Conifer	15.15	10.57	−1.81	−7.25	–	–	–	–
4	Broadleaf	5.97	4.15	−13.14	2.44	8.16	6.64	8.36	13.44
5-1	Broadleaf	6.44	10.14	−4.04	19.14	–	–	–	–
5-2	Broadleaf	8.47	11.35	0.96	14.64	–	–	–	–
6-1	Broadleaf	10.77	18.27	17.06	9.14	39.86	−37.14	–	–
6-2	Broadleaf	9.05	16.39	17.26	3.14	13.26	3.14	9.14	−42.28
7	Conifer	15.07	14.27	−24.64	−8.01	–	–	–	–
8	Broadleaf	14.12	13.64	−27.03	4.77	–	–	–	–
9	Broadleaf	8.94	8.04	−10.36	−36.14	−12.57	−40.43	–	–
10	Broadleaf	8.27	12.94	−37.85	−27.50	–	–	–	–
11	Broadleaf	11.76	20.80	22.03	−12.72	–	–	–	–
12	Broadleaf	9.41	14.40	29.52	−27.52	–	–	–	–
13	Broadleaf	12.05	17.81	39.08	10.37	–	–	–	–
14	Broadleaf	9.90	16.17	27.08	35.93	–	–	–	–
15	Broadleaf	7.65	9.93	−0.61	34.50	−41.71	16.43	–	–
16	Broadleaf	8.06	12.11	−22.58	15.23	–	–	–	–
17-1	Broadleaf	8.78	10.01	−29.84	28.82	–	–	–	–
17-2	Broadleaf	7.12	7.91	−28.71	22.43	–	–	–	–
18	Conifer	12.33	6.84	−16.50	26.40	–	–	–	–
19	Broadleaf	9.65	10.25	−14.13	33.29	−13.06	39.82	–	–
20	Broadleaf	15.64	10.65	−21.79	39.31	–	–	–	–
21	Broadleaf	5.98	3.95	−39.43	39.86	−39.16	−21.52	−19.78	1.09
22	Broadleaf	5.96	5.47	−35.29	0.43	–	–	–	–

**Table 3:** The key DIRSIG configurations of AVIRIS-C simulation.

Parameter	Value (Unit)
Scan rate	12 (Hz)
IFOV	0.8 (m rad)
Number of bands	224
Spectral range	380–2500 (nm)
Spectral sampling	10 (nm)
Spectral response	Gaussian, FWHM = 10 (nm)
Flight altitude	18.5 (km)
Flight speed	177.6 (m/s)

DIRSIG: Digital Imaging and Remote Sensing Image Generation; AVIRIS-C: “Classic” Airborne Visible Infrared Imaging Spectrometer; FWHM: Full-width-at-half-maximum.

**Table 4:** The sampling protocols and number of samples.

Interval	Number of Measurements	Number of Measurements
	in an 80m×80m Plot	in a 15m×15m Square
5 m	1360	45
10 m	720	30
15 m	400	15

**Table 5:** The *t*-test results of comparing linear models.

Pair		Slope		Intercept	
Model 1	Model 2	Test Statistic (T)	Probability (*p*)	Test Statistic (T)	Probability (*p*)
5 m-interval	10 m-interval	−0.091	0.928	0.148	0.883
5 m-interval	15 m-interval	−1.936	0.059	1.343	0.186

## References

[1] Hook S.J., Turpie K., Veraverbeke S., Wright R., Anderson M., Prakash A., Mars J., Quattrochi D. (2014). NASA 2014 The Hyperspectral Infrared Imager (HyspIRI)—Science Impact of Deploying Instruments on Separate Platforms.

[2] Yao W., van Leeuwen M., Romanczyk P., Kelbe D., van Aardt J. (2015). Assessing the impact of sub-pixel vegetation structure on imaging spectroscopy via simulation. Proc. SPIE.

[3] Watson D.J. (1947). Comparative physiological studies on the growth of field crops: I. Variation in net assimilation rate and leaf area between species and varieties, and within and between years. Ann. Bot..

[4] Chen J.M., Black T.A. (1992). Defining leaf area index for non-flat leaves. Plant Cell Environ..

[5] Lang A.R.G. (1991). Application of some of Cauchy’s theorems to estimation of surface areas of leaves, needles and branches of plants, and light transmittance. Agric. For. Meteorol..

[6] Running S.W., Ojima D. (1990). A bottom-up evolution of terrestrial ecosystem modeling. Modeling the Earth System.

[7] Bonan G.B. (1993). Importance of leaf area index and forest type when estimating photosynthesis in boreal forests. Remote Sens. Environ..

[8] Bonan G.B. (1995). Land-atmosphere interactions for climate system models: Coupling biophysical, biogeochemical, and ecosystem dynamical processes. Remote Sens. Environ..

[9] Jonckheere I., Fleck S., Nackaerts K., Muys B., Coppin P., Weiss M., Baret F. (2004). Review of methods for in situ leaf area index determination: Part I. Theories, sensors and hemispherical photography. Agric. For. Meteorol..

[10] Daughtry C.S. (1990). Direct measurements of canopy structure. Remote Sens. Rev..

[11] Chen J.M., Rich P.M., Gower S.T., Norman J.M., Plummer S. (1997). Leaf area index of boreal forests: Theory, techniques, and measurements. J. Geophys. Res. Atmos. (1984–2012).

[12] Running S.W., Nemani R.R., Peterson D.L., Band L.E., Potts D.F., Pierce L.L., Spanner M.A. (1989). Mapping regional forest evapotranspiration and photosynthesis by coupling satellite data with ecosystem simulation. Ecology.

[13] Turner D.P., Cohen W.B., Kennedy R.E., Fassnacht K.S., Briggs J.M. (1999). Relationships between leaf area index and Landsat TM spectral vegetation indices across three temperate zone sites. Remote Sens. Environ..

[14] Verrelst J., Rivera J.P., Veroustraete F., Muñoz-Marí J., Clevers J.G., Camps-Valls G., Moreno J. (2015). Experimental Sentinel-2 LAI estimation using parametric, non-parametric and physical retrieval methods—A comparison. ISPRS J. Photogramm. Remote Sens..

[15] Novelli A., Tarantino E., Fratino U., Iacobellis V., Romano G., Gentile F. (2016). A data fusion algorithm based on the Kalman filter to estimate leaf area index evolution in durum wheat by using field measurements and MODIS surface reflectance data. Remote Sens. Lett..

[16] Weiss M., Baret F., Smith G.J., Jonckheere I., Coppin P. (2004). Review of methods for in situ leaf area index (LAI) determination: Part II. Estimation of LAI, errors and sampling. Agric. For. Meteorol..

[17] AccuPAR PAR/LAI Ceptometer Model LP-80 Operator’s Manual. http://manuals.decagon.com/Manuals/10242_AccuparLP80_Web.pdf.

[18] Wilhelm W.W., Ruwe K., Schlemmer M.R. (2000). Comparison of three leaf area index meters in a corn canopy. Crop Sci..

[19] Facchi A., Baroni G., Boschetti M., Gandolfi C. (2010). Comparing opticaland direct methods for leafarea index determination in a maize crop. J. Agric. Eng..

[20] Tewolde H., Sistani K.R., Rowe D.E., Adeli A., Tsegaye T. (2005). Estimating cotton leaf area index nondestructively with a light sensor. Agron. J..

[21] Johnson M.V.V., Kiniry J.R., Burson B.L. (2010). Ceptometer deployment method affects measurement of fraction of intercepted photosynthetically active radiation. Agron. J..

[22] Hyer E.J., Goetz S.J. (2004). Comparison and sensitivity analysis of instruments and radiometric methods for LAI estimation: Assessments from a boreal forest site. Agric. For. Meteorol..

[23] Yao W., van Leeuwen M., Romanczyk P., Kelbe D., Brown S., Kerekes J., van Aardt J. Towards robust forest leaf area index assessment using an imaging spectroscopy simulation approach. Proceedings of the 2015 IEEE International Geoscience and Remote Sensing Symposium (IGARSS).

[24] Avery T.E., Burkhart H.E. (2001). Forest Measurements.

[25] Schott J.R., Brown S.D., Raqueno R.V., Gross H.N., Robinson G. (1999). An advanced synthetic image generation model and its application to multi/hyperspectral algorithm development. Can. J. Remote Sens..

[26] Schott J.R., Raqueno R.V., Salvaggio C. (1992). Incorporation of a time-dependent thermodynamic model and a radiation propagation model into IR 3D synthetic image generation. Opt. Eng..

[27] Ientilucci E.J., Brown S.D., Schott J.R., Raqueno R.V. (1998). Multispectral simulation environment for modeling low-light-level sensor systems. Proceeding of SPIE 3434, Image Intensifiers and Applications; and Characteristics and Consequences of Space Debris and Near-Earth Objects.

[28] Gartley M., Goodenough A., Brown S., Kauffman R.P. (2010). A comparison of spatial sampling techniques enabling first principles modeling of a synthetic aperture RADAR imaging platform. Proc. SPIE.

[29] Monsi M., Saeki T. (1953). Uber den Lichtfaktor in den Pflanzengesellschaften und seine Bedeutung fur die Stoffproduktion. Jpn. J. Bot..

[30] Monsi M., Saeki T. (2005). On the factor light in plant communities and its importance for matter production. Ann. Bot..

[31] Norman J.M., Jarvis P.G. (1975). Photosynthesis in Sitka spruce (Picea sitchensis (Bong.) Carr.): V. Radiation penetration theory and a test case. J. Appl. Ecol..

[32] Norman J.M., Campbell G.S., Pearcey R., Mooney H.A., Rundel P. (1989). Chapter 14: Canopy Structure. Plant Physiological Ecology: Field Methods and Instrumentation.

[33] Campbell G.S. (1986). Extinction coefficients for radiation in plant canopies calculated using an ellipsoidal inclination angle distribution. Agric. For. Meteorol..

[34] Kampe T.U., Johnson B.R., Kuester M., Keller M. (2010). NEON: The first continental-scale ecological observatory with airborne remote sensing of vegetation canopy biochemistry and structure. J. Appl. Remote Sens..

[35] Kampe T., Leisso N., Musinsky J., Petroy S., Karpowicz B., Krause K., Crocker R.I., DeVoe M., Penniman E., Guadagno T. (2013). TM-005: The NEON 2013 Airborne Campaign at Domain 17 Terrestrial and Aquatic Sites in California. http://www.neoninc.org/data-resources/papers-publications/.

[36] Onyx Computing, Inc. OnyxTREE [Software]. 1992–2011. http://www.onyxtree.com.

[37] Jones B.W. (2007). Discovering the Solar System.

[38] Reda I., Andreas A. (2004). Solar position algorithm for solar radiation applications. Sol. Energy.

[39] Berk A., Anderson G.P., Bernstein L.S., Acharya P.K., Dothe H., Matthew M.W., Adler-Golden S.M., Chetwynd J.H., Richtsmeier S.C., Pukall B. (1999). MODTRAN4 radiative transfer modeling for atmospheric correction. Proc. SPIE.

[40] Richardson A.J., Weigand C.L. (1977). Distinguishing vegetation from soil background information. Photogramm. Eng. Remote Sens..

[41] Gong P., Pu R., Biging G.S., Larrieu M.R. (2003). Estimation of forest leaf area index using vegetation indices derived from Hyperion hyperspectral data. IEEE Trans. Geosci. Remote Sens..

[42] Lüdeke M., Janecek A., Kohlmaier G.H. (2002). Modelling the seasonal CO_2_ uptake by land vegetation using the global vegetation index. Tellus B.

[43] Wang Q., Adiku S., Tenhunen J., Granier A. (2005). On the relationship of NDVI with leaf area index in a deciduous forest site. Remote Sens. Environ..

[44] Kleinbaum D.G., Kupper L.L. (1978). Applied Regression Analysis and Other Multivariable Methods.

[45] Pagano M., Gauvreau K. (2000). Principles of Biostatistics.

[46] Oberkampf W.L., DeLand S.M., Rutherford B.M., Diegert K.V., Alvin K.F. (2002). Error and uncertainty in modeling and simulation. Reliab. Eng. Syst. Saf..

